# Supplementary material for: Impact of multiple waves of COVID-19 on healthcare networks in the United States
Source: PLoS One. 2021 Mar 3;16(3):e0247463. doi: 10.1371/journal.pone.0247463 (PMC7929642; doi:10.1371/journal.pone.0247463)
Supplement: S1 File — (DOCX) [file pone.0247463.s001.docx]

Impact of Multiple Waves of COVID-19 on Healthcare Networks in the United States

Emad M. Hassan, Hussam Mahmoud*****

Department of Civil and Environmental Engineering, Colorado State University, Fort Collins, CO, USA

Email: [Hussam.Mahmoud@colostate.edu](mailto:Hussam.Mahmoud@colostate.edu)

Tel: 970-491-6605

**This file includes:**

Figs S1 to S10

Tables S1

**Fig S1** shows the distribution of total beds, inpatient beds, ICU beds, and ICU beds with mechanical ventilators before the occurrence of the COVID-19 pandemic. Many counties are shown to have no staffed beds and others have a lesser number of staffed beds than the national average.


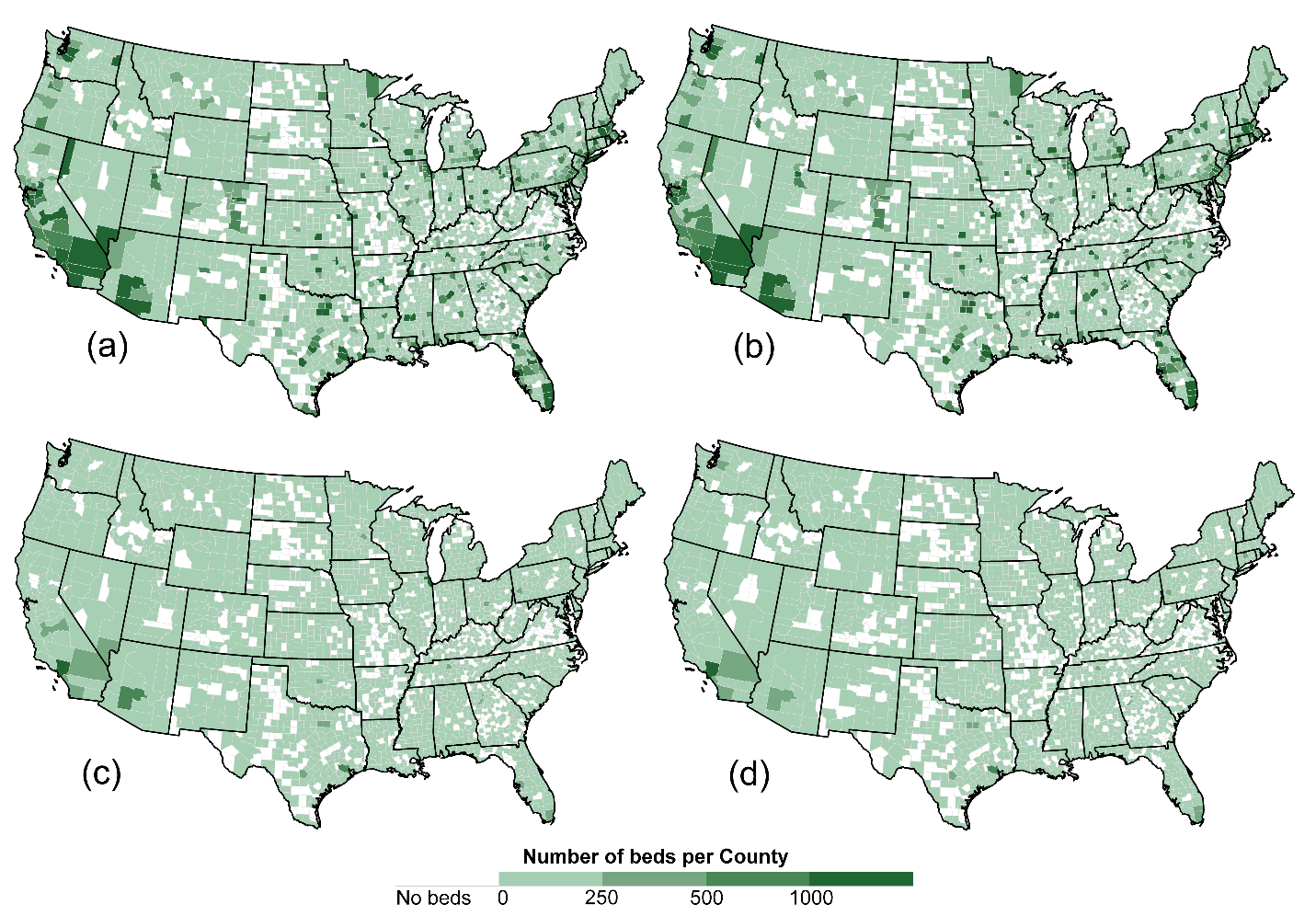


**Fig S1.** Distributions of the total number of unoccupied beds per county before the pandemic that can be used by the COVID-19 patients. **a**) total beds, **b**) Inpatient beds, **c**) ICU beds, and **d**) ICU beds with mechanical ventilators. These distributions are calculated based on data extracted from the National Healthcare Safety Network (NHSN) at the beginning of the pandemic outbreak in the United States [1].

**Figure S2** displays the population distribution in the United States where Figure S2 (a) and Figure S2 (b) show the total and vulnerable (aged +60) populations, respectively. The figure shows some counties to have a higher number of vulnerable residents and may require increased levels of protection measures.


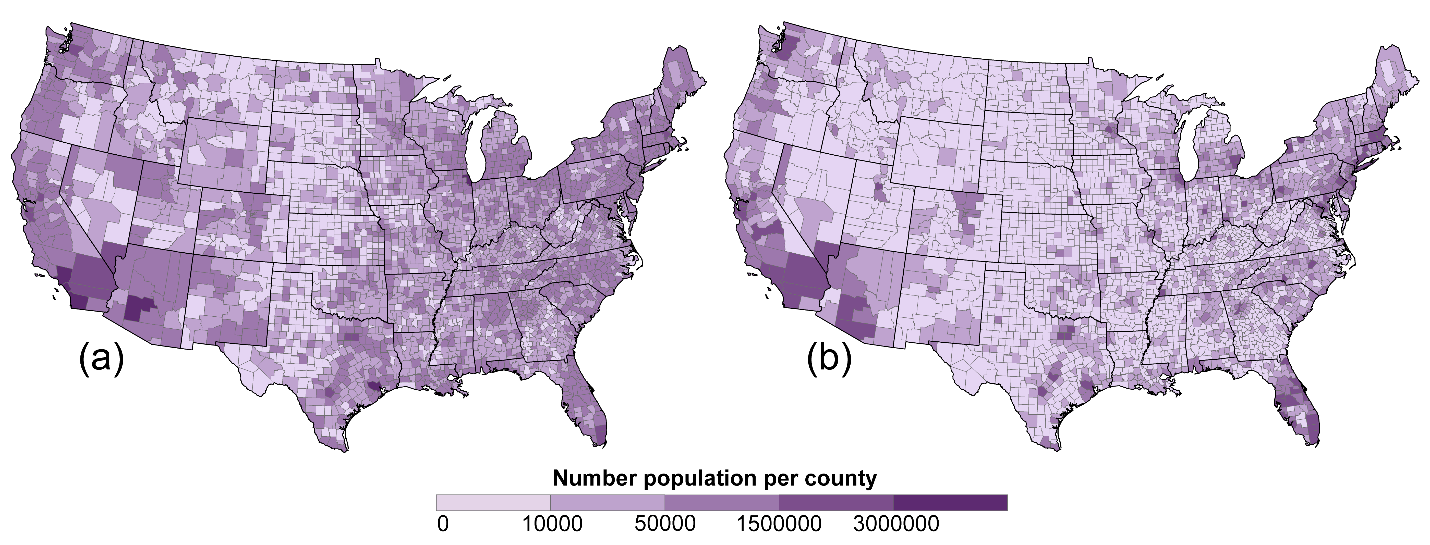


**Fig S2.** Population distribution in the U.S. by county. **a)** total and **b)** residents aged +60 [2].

**Table S1**. Ending dates for the stay-at-home order per state, reduction in mobilization described as the percentage of population staying home, mask mandatory order, and the status of schools and workplaces with respect to reopening and closing.

| State | stay-at-home order ending date [3] | % Population staying at home [4] | Masks mandatory order [3] | School reopening order [5] | Workplace reopening state [3] |
| --- | --- | --- | --- | --- | --- |
| Alabama | 4/30/2020 | 23.1 | Mandatory | No order | Mostly open |
| Alaska | 4/21/2020 | 33.1 | No restrictions | No order | Mostly open |
| Arizona | 5/15/2020 | 30.6 | Sometimes | No order | Mixed |
| Arkansas | 5/8/2020* | 23.1 | Sometimes | Ordered open | Mostly open |
| California | 6/15/2020* | 32.2 | Mandatory | Partial closure | Mostly closed |
| Colorado | 5/8/2020 | 32.4 | Mandatory | No order | Mixed |
| Connecticut | 5/20/2020 | 29.3 | Mandatory | No order | Mixed |
| Delaware | 5/15/2020 | 27.9 | Mandatory | Partial closure | Mostly open |
| D.C. | 5/29/2020 | 31.1 | Mandatory | Full closure | Mostly open |
| Florida | 4/30/2020 | 27.3 | No restrictions | Ordered open | Mostly open |
| Georgia | 4/30/2020 | 25.8 | No restrictions | No order | Mostly open |
| Hawaii | 5/31/2020 | 29.4 | Mandatory | Partial closure | Mixed |
| Idaho | 4/30/2020 | 28.5 | No restrictions | No order | Mixed |
| Illinois | 5/30/2020 | 30.1 | Mandatory | No order | Mostly closed |
| Indiana | 5/1/2020 | 27.5 | Mandatory | No order | Mostly open |
| Iowa | 5/8/2020* | 28.1 | Mandatory | Ordered open | Mostly open |
| Kansas | 5/3/2020 | 29.3 | Sometimes | No order | Mostly open |
| Kentucky | 5/22/2020* | 28.0 | Mandatory | No order | Mostly closed |
| Louisiana | 5/14/2020 | 25.0 | Sometimes | No order | Mixed |
| Maine | 5/31/2020 | 29.1 | Mandatory | No order | Mixed |
| Maryland | 5/15/2020 | 28.1 | Mandatory | No order | Mostly open |
| Massachusetts | 5/18/2020 | 32.1 | Mandatory | No order | Mixed |
| Michigan | 5/15/2020 | 29.3 | Mandatory | No order | Mostly closed |
| Minnesota | 5/4/2020 | 29.7 | Mandatory | No order | Mostly closed |
| Mississippi | 5/11/2020 | 21.0 | Mandatory | No order | Mostly open |
| Missouri | 5/3/202 | 29.1 | No restrictions | No order | Mostly open |
| Montana | 4/24/2020 | 31.8 | Mandatory | No order | Mostly open |
| Nebraska | 5/4/2020* | 28.2 | Sometimes | No order | Mostly open |
| Nevada | 5/15/2020 | 31.7 | Mandatory | No order | Mostly open |
| New Hampshire | 5/4/2020 | 27.3 | Mandatory | No order | Mostly open |
| New Jersey | 6/15/2020* | 31.4 | Mandatory | No order | Mostly open |
| New Mexico | 5/15/2020 | 29.1 | Mandatory | No order | Mostly closed |
| New York | 5/15/2020 | 35.4 | Mandatory | Partial closure | Mixed |
| North Carolina | 5/8/2020 | 26.0 | Mandatory | Partial closure | Mixed |
| North Dakota | 5/1/2020* | 28.0 | Mandatory | Partial closure | Mostly open |
| Ohio | 5/30/2020 | 28.9 | Mandatory | No order | Mostly open |
| Oklahoma | 5/15/2020* | 27.7 | No restrictions | No order | Mostly open |
| Oregon | 5/15/2020* | 32.3 | Mandatory | No order | Mostly closed |
| Pennsylvania | 5/8/2020 | 29.9 | Mandatory | Partial closure | Mostly open |
| Rhode Island | 5/8/2020 | 29.5 | Mandatory | No order | Mostly closed |
| South Carolina | 5/12/2020 | 24.6 | Sometimes | Full closure | Mostly open |
| South Dakota | 5/15/2020* | 26.0 | No restrictions | No order | Mostly open |
| Tennessee | 4/30/2020 | 25.3 | No restrictions | No order | Mostly open |
| Texas | 4/30/2020 | 24.9 | Mandatory | No order | Mostly open |
| Utah | 5/16/2020* | 30.3 | Mandatory | No order | Mostly open |
| Vermont | 5/15/2020 | 28.5 | Mandatory | Ordered open | Mixed |
| Virginia | 6/10/2020 | 27.5 | Mandatory | No order | Mostly open |
| Washington | 5/4/2020 | 30.3 | Mandatory | No order | Mostly closed |
| West Virginia | 5/4/2020 | 28.5 | Mandatory | No order | Mostly open |
| Wisconsin | 5/26/2020 | 28.1 | Mandatory | No order | Mostly open |
| Wyoming | 5/15/2020* | 29.4 | No restrictions | Partial closure | Mostly open |

* The stay-at-home-order was never issued, extended, or expired, and mobility data are used to estimate the reopening date.

**Fig S3** shows the impact of the multiple waves COVID-19 pandemic on the distribution of cases that need hospitalization services and counties that are expected to have a demand exceeding their hospital facilities capacity. This figure demonstrates that the expected third wave even without easing the current protective measured applied by each county, will cause many of the counties located in the middle and southern part of the U.S. to be overwhelmed with pandemic-related patients.


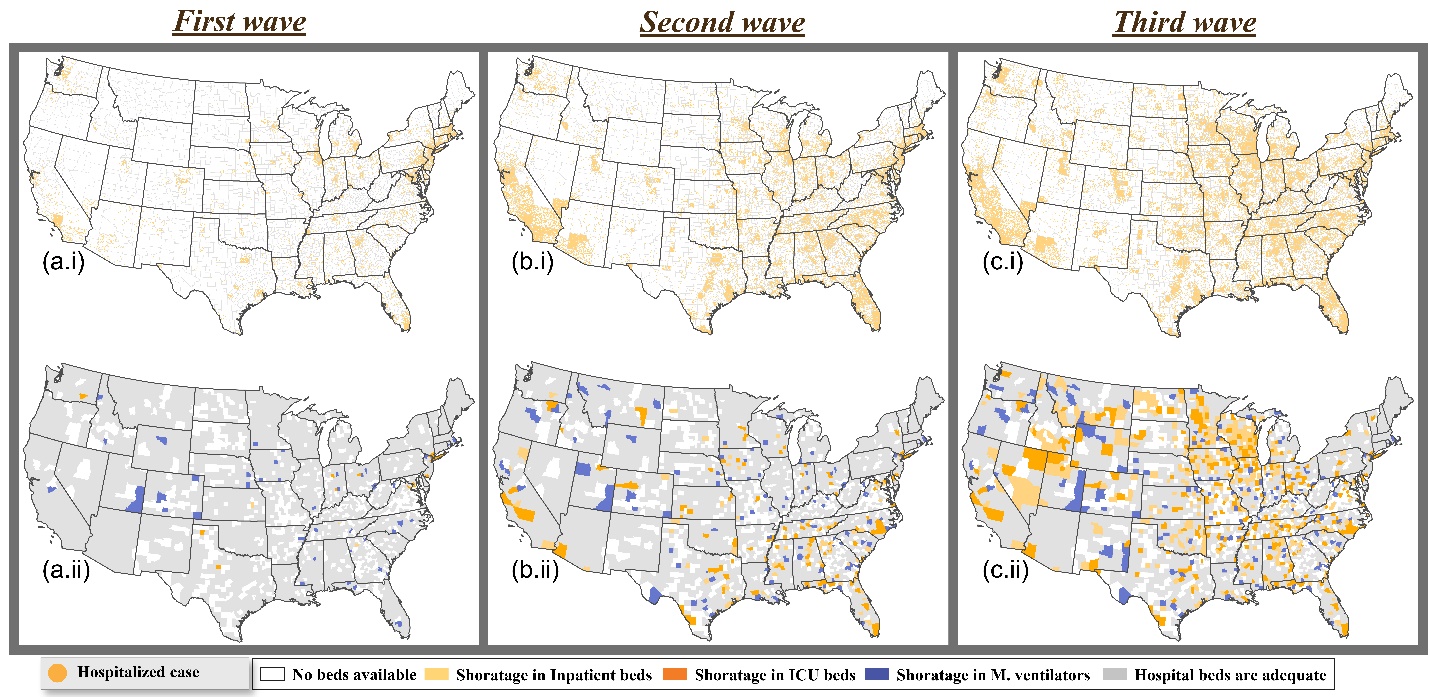


**Fig S3.** Impact of COVID-19 multiple waves a) first wave, b) second wave, and c) third wave on i) the distribution of the peak number of hospitalized cases and the distribution of counties with overwhelmed inpatient beds, ICU beds, and mechanical ventilators.

**Fig S4**, **Fig S5**, and **Fig S6** display a comparison between the expected number of COVID-19 cases who need different hospitalization services and the capacity of the hospital facilities in each state. Different scenarios are utilized including basic case (no easing in the applied protective measures), full susceptible population and 50% protection rate, and fully susceptible population and no protection.


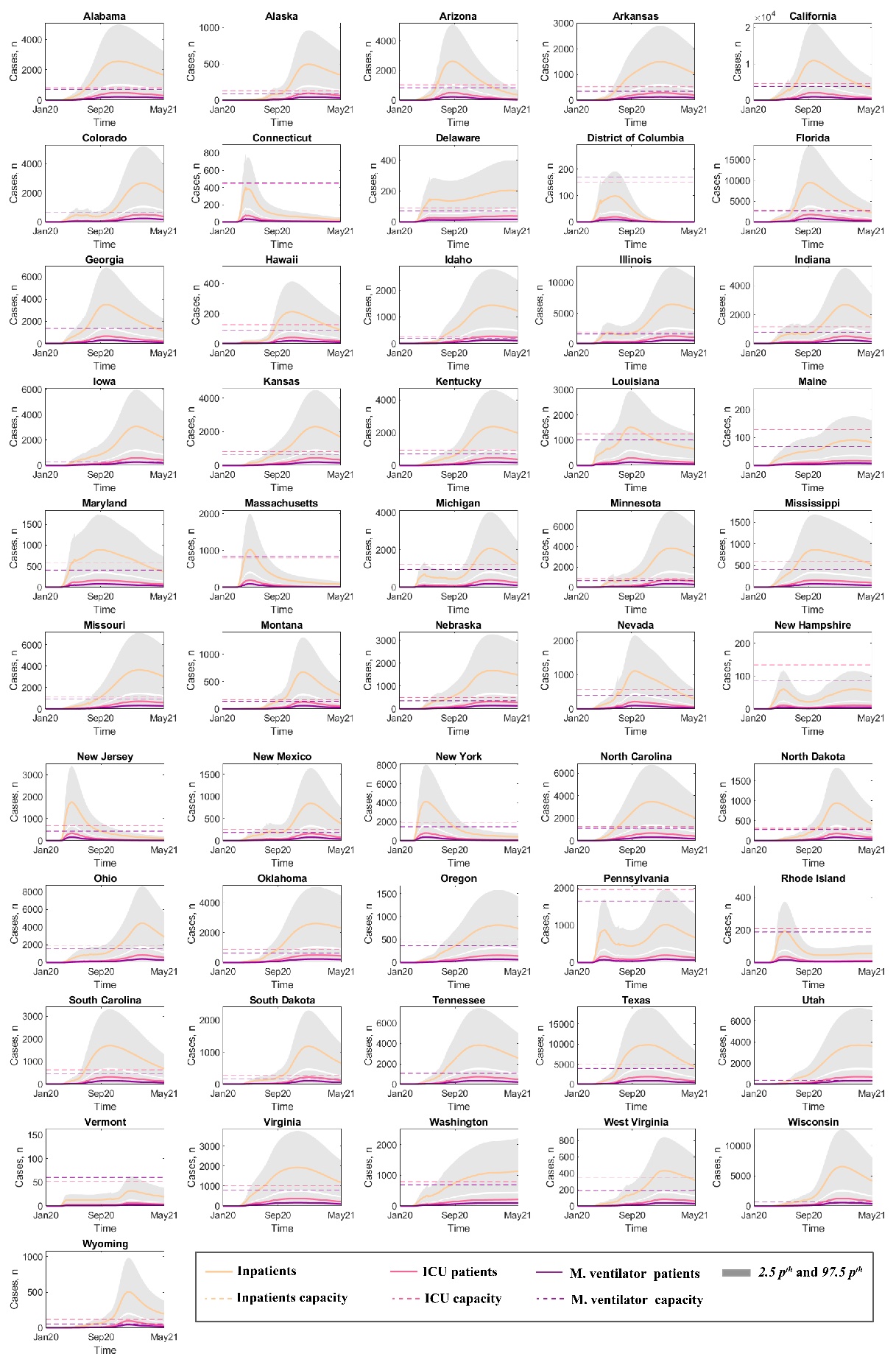


**Fig S4.** Expected number of hospitalized cases per state, including inpatients, ICU patients, and mechanical ventilator patients, for the case of no change in the number of susceptible cases (*S*) or the protection rate (*α*).


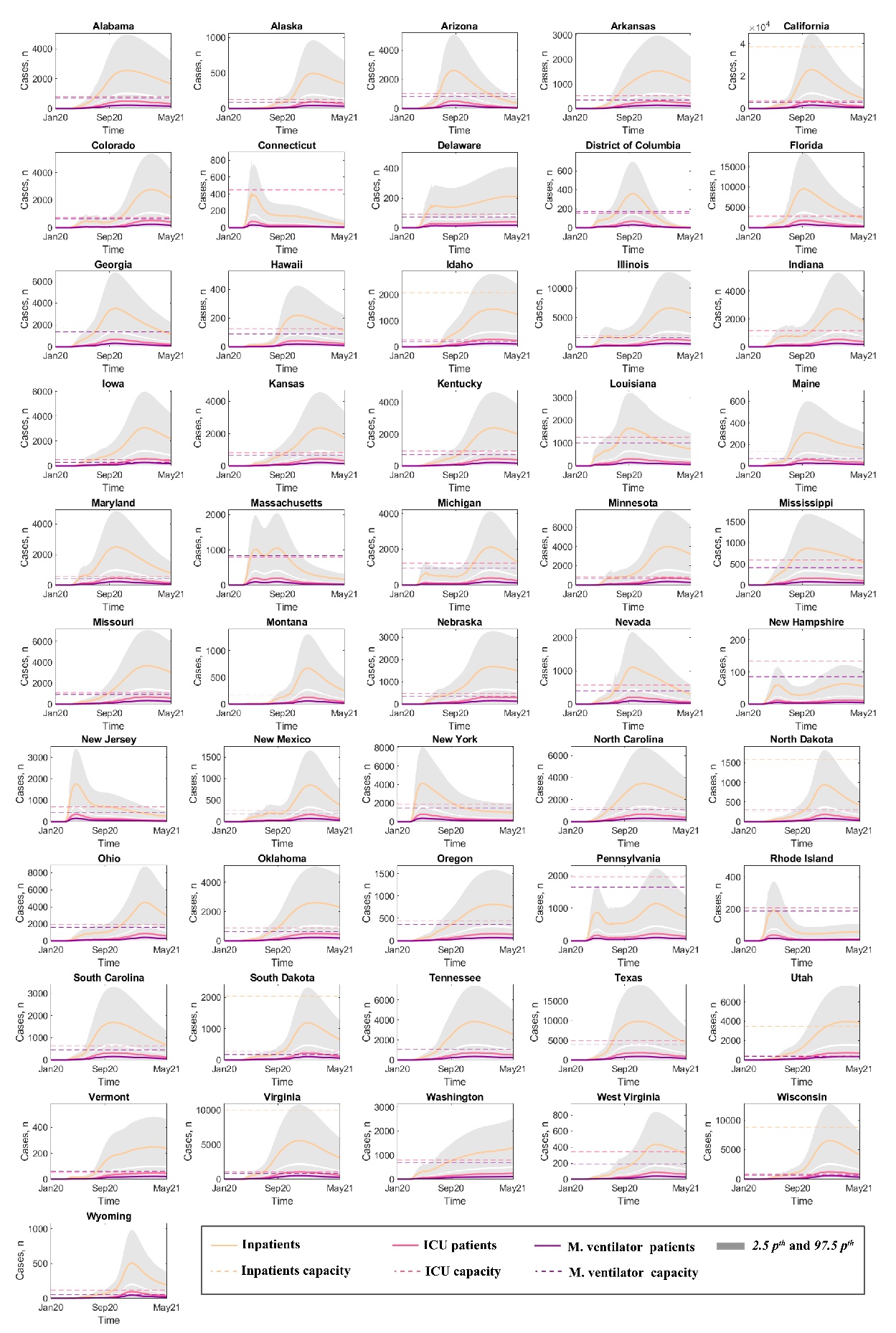


**Fig S5.** Expected number of hospitalized cases per state, including inpatients, ICU patients, and mechanical ventilator patients, for the case of ∆*S* = 1 and ∆*α* = 0.5.


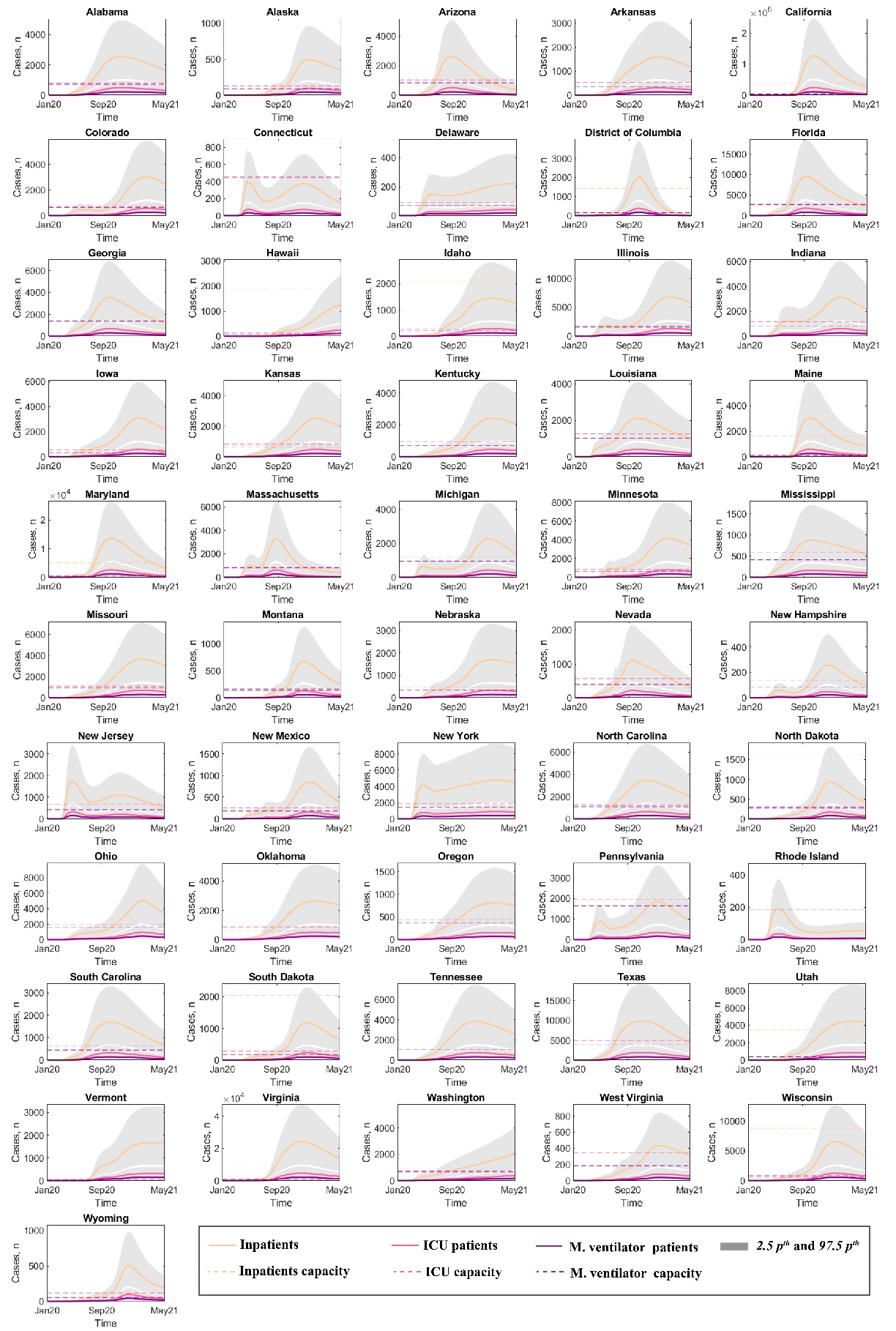


**Fig S6.** Expected number of hospitalized cases per state, including inpatients, ICU patients, and mechanical ventilator patients, for the case of ∆*S* = 1 and ∆*α* = 0.

**Fig S7** displays the distribution of counties with hospitals that are expected to be overwhelmed for different state reopening scenarios.


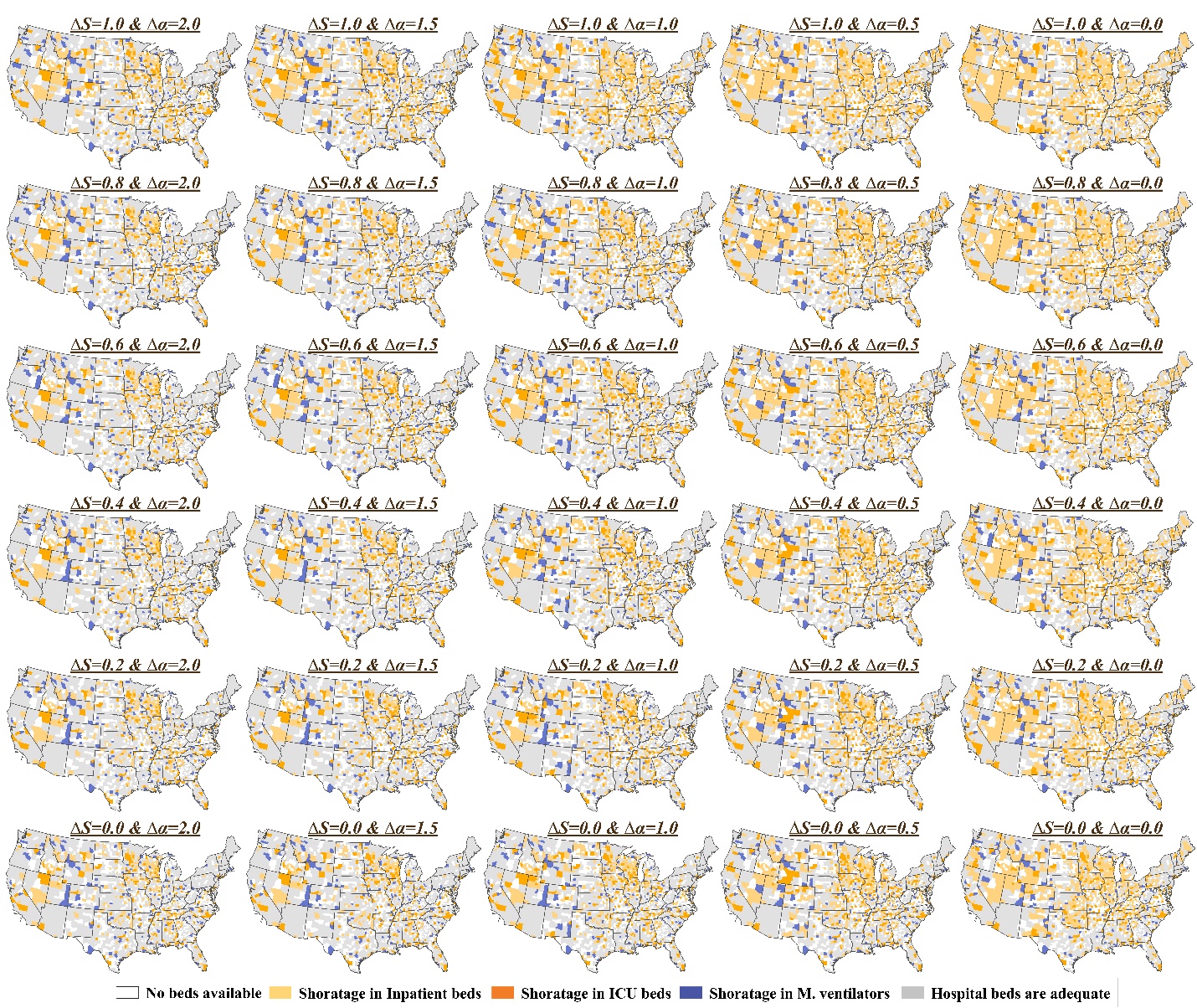


**Fig S7.** Distribution of the counties that expected to be overwhelmed with the COVID-19 patients during peak cases based on different susceptible cases and protection rates.

Different countries are applying various protective measures to limit the spread of the COVID-19 virus and also different mitigation strategies to reduce the overwhelming of the healthcare system. While the first outbreak of COVID-19 occurred in China, the applied lockdown and tracking of the exposed cases allowed the country to relax the initially applied strong measures and reduce the need for the mobile hospitals constructed to reduce the healthcare burden. Germany is another country that successfully reduces the number of infected and hospitalized cases, where the healthcare system successfully accommodates the surge of COID-19 related patients. India is another example where the healthcare system is partially overwhelmed; due to the fact that it has one of the lowest numbers of hospital staffed beds per capita. **Fig S8** shows a comparison between the expected number of COVID-19 cases at different countries for the basic case (no easing in the applied protective measures) and full susceptible population and 50% protection rate. This figure shows a higher risk of demand on the hospital facilities exceeding the capacity if an easing of protective measures is applied in countries such as India. While a high magnitude wave is expected at countries that managed their disease spread such as Germany and China.


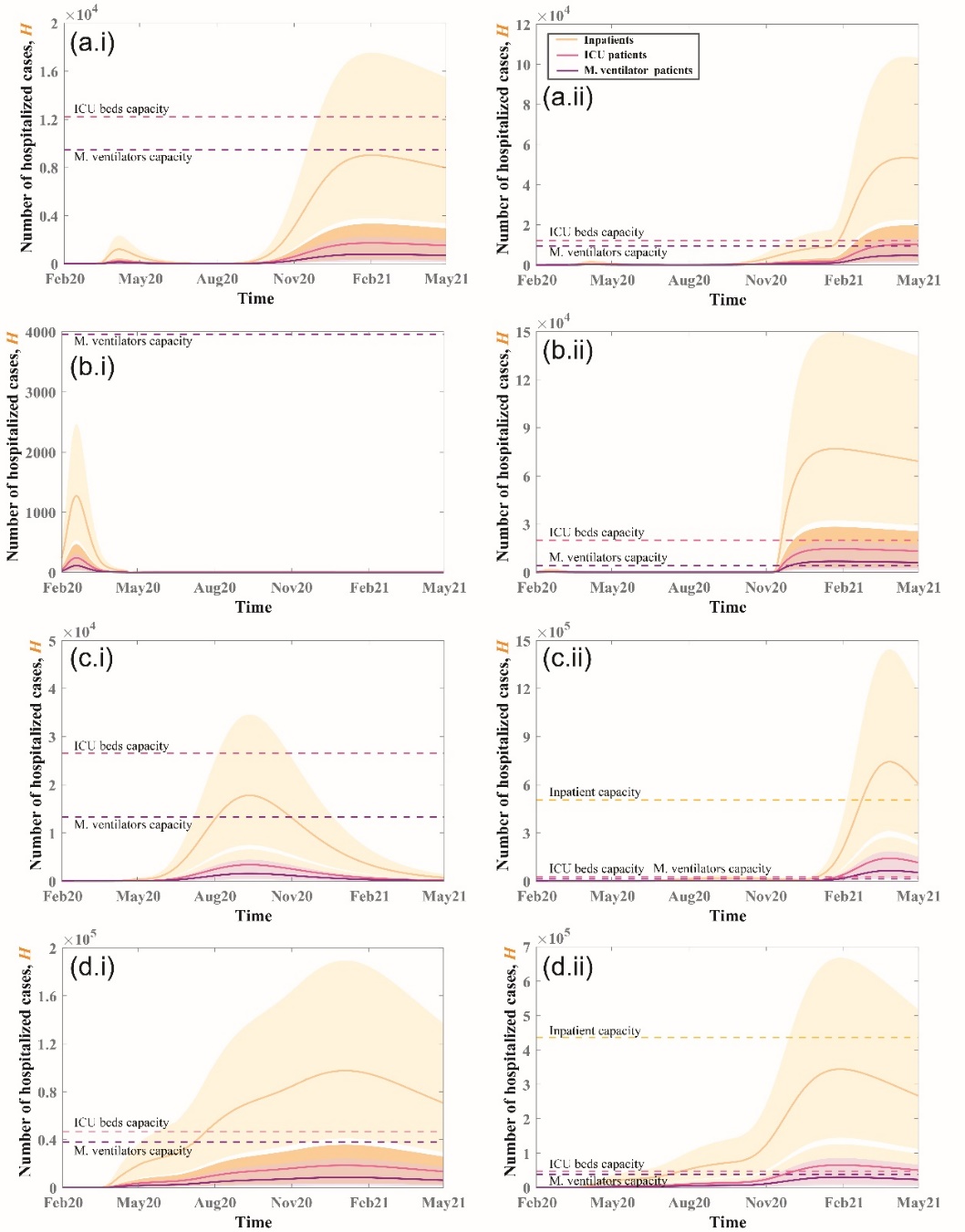


**Fig S8.** The expected number of hospitalization cases for different countries including **a**) Germany, **b**) China, **c**) India, and **d**) U.S compared with the hospital facilities capacity for i) the basic case and ii) the case of *∆S* = 1 and *∆α* = 0.5.

**Fig S9** presents the effect of different lengths of state lockdown on the peak of hospitalization cases and the number of overwhelming hospital facilities in the US counties. The case of full susceptible population and 50% protection rate is utilized. The figure shows that even a short period of lockdown can significantly reduce the peak of the expected third wave.


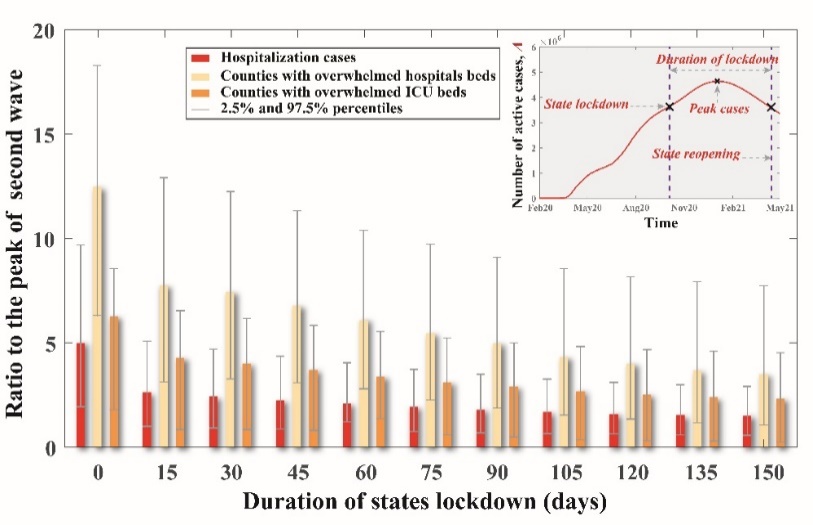


**Fig S9.** Impact of states lockdown period on the ratio of the peak number of hospitalization cases, counties with overwhelmed hospital beds, and counties with overwhelmed ICU beds to the second wave.

**Fig S10** displays the distribution of the required additional hospital beds and mechanical ventilators per state to overcome the expected overwhelming that resulted from easing the applied protective measures while increasing or maintaining the current susceptible cases. The figure shows that most of the Mid-America states will need additional beds and mechanical ventilators if easing the applied protective measures take place.


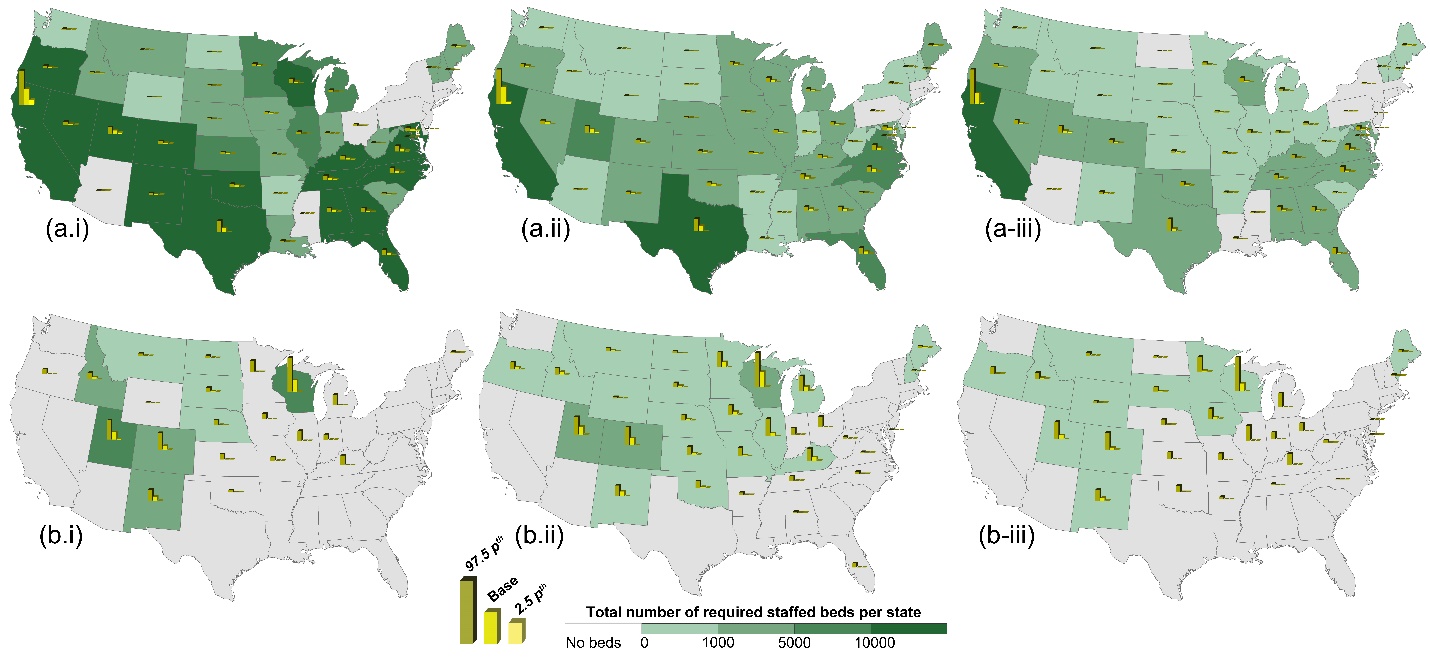


**Fig S10.** Required number of additional staffed beds per state to avoid the hospital facilities overwhelmed for **a**) the case of ∆*S* = 1 and ∆*α* = 0 and **b**) the case of ∆*S* = 0 and ∆*α* = 0 based on **i**) inpatient beds, **ii**) ICU beds, and **iii**) mechanical ventilators.

References:

1. National Healthcare Safety Network (NHSN). COVID-19 Module Data Dashboard – Overview. 2020 [cited 6 Nov 2020]. Available: https://www.cdc.gov/nhsn/covid19/report-overview.html

2. U.S. Census Bureau. American Community Survey 1-year estimates. In: 2019 [Internet]. [cited 6 Nov 2020]. Available: http://censusreporter.org/profiles/01000US-united-states/

3. The New York Times. See Coronavirus restrictions and mask mandates for all 50 states. 2020 [cited 6 Nov 2020]. Available: https://www.nytimes.com/interactive/2020/us/states-reopen-map-coronavirus.html

4. Bureau of Transportation Statistics. Daily travel during the COVID-19 public health emergency. 2020 [cited 6 Nov 2020]. Available: https://www.bts.gov/browse-statistical-products-and-data/trips-distance/explore-us-mobility-during-covid-19-pandemic

5. Education Week. Map: Where are schools closed? 2020 [cited 6 Nov 2020]. Available: https://www.edweek.org/leadership/map-where-are-schools-closed/2020/07
