# Supplementary material for: Impact of multiple waves of COVID-19 on healthcare networks in the United States
Source: PLoS One. 2021 Mar 3;16(3):e0247463. doi: 10.1371/journal.pone.0247463 (PMC7929642; doi:10.1371/journal.pone.0247463)
Supplement: Attachment — Submitted filename: Response to Reviewers.docx [file pone.0247463.s002.docx]

**Impact of Multiple Waves of COVID-19 on Healthcare Networks in the United States**

**RESPONSE TO JOURNAL REQUIREMENT COMMENTS:**

1. Thank your detailed response to our copyright questions. The data from government sources (US Census and USA facts) is compatible for publication. However, as Definitive Healthcare allows non-commercial use, this is not compatible. All PLOS content is published under the Creative Commons Attribution (CC BY) 4.0 license, which means that they will be freely available online, and any third party is permitted to access, download, copy, distribute, and use these materials in any way, even commercially. We require one of the following:

- Specific consent from the copyright holder to publish these images in PLOS ONE, under the CC BY 4.0 license. Please include a file including the granted permission as a Supporting Information file and include the following text within your figure caption(s): “Republished from [ref] under a CC BY license, with permission from [name of publisher], original copyright [original copyright year].”
- The authors remove these images from their submission.

**Authors Response:** Thank you for the follow regarding the figures. To avoid any issues with the license related to data from Definitive Healthcare, we replaced Figure 1 (d), which was the only figure that directly represents data from Definitive Healthcare, with another figure using data from Homeland Infrastructure Foundation-Level Data (HIFLD). For clarifications, Figure 1 (e) and (f) and Figure S1 in the supporting information, as well as all other figures in the manuscript, are all produced by the authors and do not show nor represent any data directly collected from Definitive Healthcare.

The HIFLD respective file (shapefile) contains the following note about the License: “None (Public Use)” and all the HIFLD data are open source as noted: “This site provides National foundation-level geospatial data within the open public domain that can be useful to support community preparedness, resiliency, research, and more. The data is available for download as CSV, KML, Shapefile, and accessible via web services to support application development and data visualization.”

1. Homeland Infrastructure Foundation-Level Data (HIFLD):

https://hifld-geoplatform.opendata.arcgis.com/datasets/hospitals?geometry=94.405%2C-16.829%2C-125.321%2C72.120
